# Sexual Dimorphism of Size Ontogeny and Life History

**DOI:** 10.3389/fped.2020.00387

**Published:** 2020-07-24

**Authors:** Alina German, Ze'ev Hochberg

**Affiliations:** ^1^Pediatric Department, Bnei-Zion Medical Center, Clalit HMO, Haifa, Israel; ^2^Rappaport Family Faculty of Medicine, Technion—Israel Institute of Technology, Haifa, Israel

**Keywords:** sexual size dimorphism, male/female height ratio, hypoallometry, hyperallometry, environmental stress, subsistence-based societies, GDP per capita, life expectancy

## Abstract

**Background:** Ecological and physiological factors and social and economic constraints affect sex-specific body size. Here, we used the male/female (M/F) height ratio as an indicator of the combined effect of genetic and sex characteristics. We hypothesized that (1) sexual dimorphism in body size will be established during infancy and adolescence when growth velocity is maximal, (2) living standards and health are important factors which can affect sexual dimorphism in body size, (3) variations in sexual dimorphism in body size are due to the differential response of boys and girls to environmental cues, and (4) sexual dimorphism in body size will be more pronounced in those populations whose average height and weight are the greatest.

**Methods:** To study the ontogeny of sexual dimorphism from birth until the age of 18 years, we used the 2000 CDC growth data. Data on height by country, life expectancy, and gross domestic product (GDP) per capita based on purchasing power parity were extracted from the national accounts data of NCD Risk Factor Collaboration, the World Bank, Eurostat: Demographic Statistics, Secretariat of the Pacific Community: Statistics and Demography Program, and the US Census Bureau.

**Results:** We found that sexual dimorphism in body size starts at age 1 month, peaks at age 3 months, and diminishes by age 24 months. During childhood, there is no sexual difference in body size, and it is gradually established when the boys enter puberty. The M/F height ratio correlates positively with the average male and female height and weight by country.

**Conclusion:** Sexual dimorphism in body size occurs when (a) the growth velocity is maximal during infancy and adolescence, (b) living standards are high, and health correlate positively with male/female height ratio. Anthropological studies and our results emphasize mostly the female resiliency hypothesis: shorter male heights in times of environmental stress lead to smaller sexual dimorphism in body size.

For some animal species, sexual, survival, and fecundity selection can influence the degree of sexual dimorphism in body size (1, 2). In humans, ecological and physiological factors and social and economic constraints may also affect sex-specific body size (3, 4) The relationship between male and female body size is hyperallometric in those taxa where males are larger and hypoallometric in those taxa where females are larger (5). In humans, different hypotheses have been proposed to explain the variation of sexual dimorphism in height, but no consensus has emerged. It has been proposed that the extent of sexual dimorphism in human populations results from a trade-off between size-related mortality and size-related obstetric complications and fertility (2). A study of dental data for great apes and ten hominid samples reported a decline in sexual dimorphism in body size during the last three million years (6). There is a contrasting report that contemporary agriculturalists exhibit a greater degree of sexual dimorphism in stature than present-day hunter-gatherers (7). Sociologists argue that sex differences in stature between societies can be influenced by female discrimination, female labor participation, and relative mobility and the effects of famine and crisis periods (3, 4).

Sexual dimorphism in body size may serve strategic evolutionary fitness goals, which are also the outcome of the different responses of boys and girls to environmental cues. Stature variation among populations results from a complex interaction of genetic and environmental influences. Two genotypes that can produce the same adult height under optimal environmental circumstances can also produce different heights under circumstances of deprivation (3). Thus, children who are tall in a wealthy community might be short when the economic conditions are poor. Height is generally believed to be mostly influenced by the quality and quantity of nutrition and the disease environment, to the extent that some economic historians have used height as a measure of a population's living standards (8–10). The male/female (M/F) height ratio can serve as an indicator of the combined effect of genetic and sex characteristics and the social and economic environment. Since stature is most vulnerable in youth, especially during the first year of life (11), analyzing adult heights can indicate the susceptibility of children to their environment.

Against this background, we hypothesized that (1) sexual dimorphism in body size will be established during infancy and adolescence when growth velocity is maximal, (2) living standards and health are important factors which can affect sexual dimorphism in body size, (3) variations sexual dimorphism in body size are due to the differential response of boys and girls to environmental cues, and (4) sexual dimorphism in body size will be more pronounced in those populations whose average height and weight are the greatest.

In order to understand the impact of living standard and health on sexual dimorphism in body size, we correlated the gross domestic product (GDP) per capita based on purchasing power parity and life expectancy (survival selection) with the sexual dimorphism in body size in both 161 modern countries and subsistence-based preindustrial societies. We considered a country and a society to be a population of people that is related by geographical context and by descent, but also by its physical cultural and economic environment. A population so understood is a temporally continuous, spatially scattered entity that changes over time (12). We used the average male and female height and weight by country from 1992 to 1996 as obtained from the NCD Risk Factor Collaboration (NCD-RisC) dataset of average country heights from 1896 to the present day (http://www.ncdrisc.org/d-height.html) and a database of 34 preindustrial societies.

## Methods

### Data Sources

To study the ontogeny of sexual dimorphism on male and female height from birth until the age of 18 years, we used the 2000 CDC growth data http://www.cdc.gov/growthcharts/.

GDP per capita is an economic snapshot of a country and was used to estimate the country's economic health. Data on GDP per capita ($US) based on purchasing power parity were extracted from the national accounts data of the World Bank (http://data.worldbank.org/indicator/NY.GDP.PCAP.CD) and entered into the database.

Life expectancy at birth indicates the number of years a newborn infant would live if prevailing patterns of mortality at the time of birth were to stay the same throughout life and was used as a measure of health. There are great variations in life expectancy between different parts of the world, and these differences are mostly caused by differences in public health, medical care, and diet. The data on life expectancy at birth were collected from the World Bank's data bank, whose sources are (a) the United Nations (UN) Population Division, World Population Prospects, (b) the United Nations Statistical Division, Population and Vital Statistics Report (various years), (c) census reports, and other statistical publications from national statistical offices, (d) Eurostat: Demographic Statistics, (e) Secretariat of the Pacific Community: Statistics and Demography Program, and (f) the US Census Bureau: International Database (http://data.worldbank.org/indicator/SP.DYN.LE00.IN) and entered into the database.

To determine whether sexual dimorphism in body size will be greater in countries whose population's average height and weight are greater than those in countries whose population's average height and weight are smaller, we used data on average male and female height by country in 161 countries from 1992 to 1996. These data were obtained with permission from the NCD Risk Factor Collaboration (NCD-RiSC) dataset (http://www.ncdrisc.org/d-height.html) and entered into the database. This dataset includes sources that were representative of a national, subnational, or community population and had measured height. Self-reported height and data sources on population subgroups whose anthropometric status may differ systematically from that of the general population were not included in the study. Size dimorphism is defined as the ratio between male and female height.

Data on 34 subsistence-based societies was obtained from Robert Walker's unpublished open source field notes (http://anthropology.missouri.edu/people/walker.html) and his published reports (13). The data on population density, which in subsistence-based society represents the economic wealth in a positive correlation, the average male and female height, body weight, life expectancy at birth, and at the age of 15 years was entered into the dataset. At the time of data collection on the subsistence-based societies, ethical approval was not required, and the authors had no access to any individual's data.

### Statistical Analysis

All statistical analyses were done using a software statistical package (IBM SPSS Statistics 20.0) and statistical significance was set as 5%. The M/F height ratio as a function of age was calculated using growth data from birth until age 18 years. The Pearson's correlation coefficient was used to calculate the strength and direction of association of the linear relationship between the M/F height ratio by country and the average anthropometric parameters of males and females from 161 countries, GDP, and life expectancy. The Spearman's correlation coefficient was used as a nonparametric measure of the strength and direction of association between the M/F height ratio and the average anthropometric parameters of males and females, life expectancy, population density in preindustrial societies.

## Results

**Figure 1:**
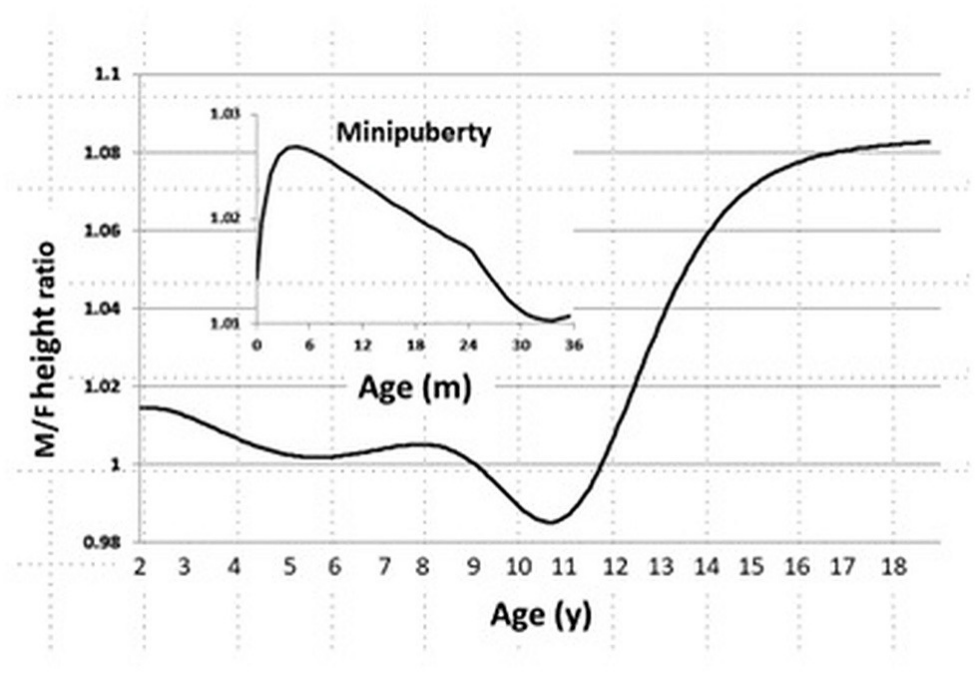
Sexual dimorphism in body size from age 0 to 20 years in the CDC 2000 database. Male/Female height ratio as a function of age. Minipuberty occurs mainly in the first 3–6 months of life in both sexes when there is transient activation of the hypothalamic-pituitary-gonadal axis during the neonatal period and results in high serum gonadotropin and sex steroid hormone levels.

In the CDC 2000 growth database, we found that sexual dimorphism in body size starts at age 1 month, peaks at age 3 months, and declines by age 24 months. During childhood, we also found no sexual difference in body size, and it is gradually established when the boys enter puberty (Figure 1).

**Figure 2:**
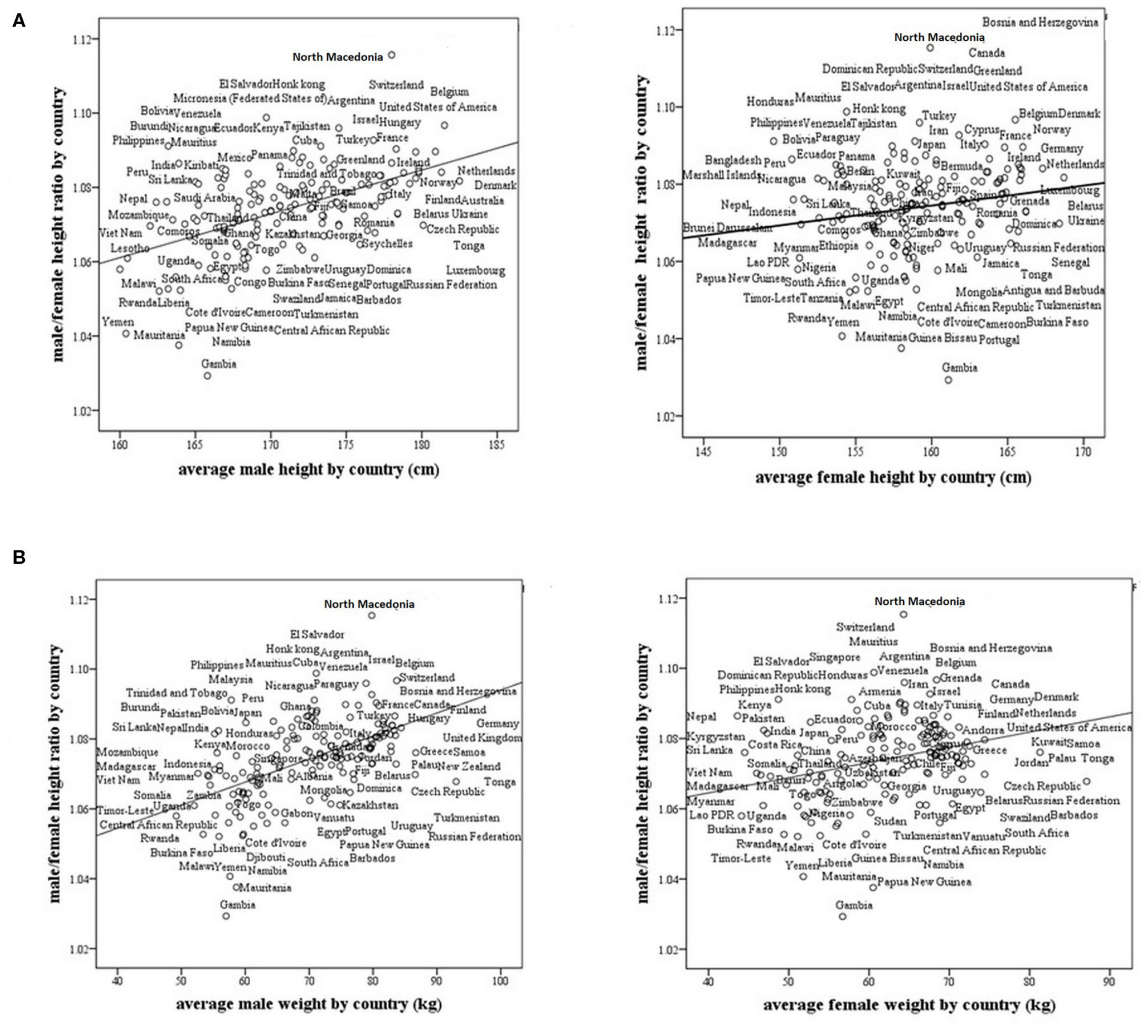
Male/female height ratio as a function of the average male and female size in 161 modern countries. **(A)** Male/female height ratio as a function of the average male and female height by country. For males, *R* = 0.519, *p* < 0.0001, and for females, *R* = 0.18, *p* = 0.019. **(B)** Male/female height ratio as a function of the average male and female weight by country. For males, *R* = 0.54, *p* < 0.0001 and for females, *R* = 0.29, *p* < 0.0001. R-Pearson's correlation coefficient.

We found that the M/F height ratio correlates positively and strongly with the average male height by country (*r* = 0.519, *p* < 0.0001). The correlation between the M/F height ratio and the average female height by country was also positive, but not as strong as that for the average male height by country (*r* = 0.18, *p* = 0.019, Figure 2A). The correlation between the M/F height ratio and male weight (*r* = 0.54, *p* < 0.0001) was also positive and stronger than that between the M/F height ratio and female weight (*r* = 0.29, *p* < 0.0001, Figure 2B).

**Figure 3:**
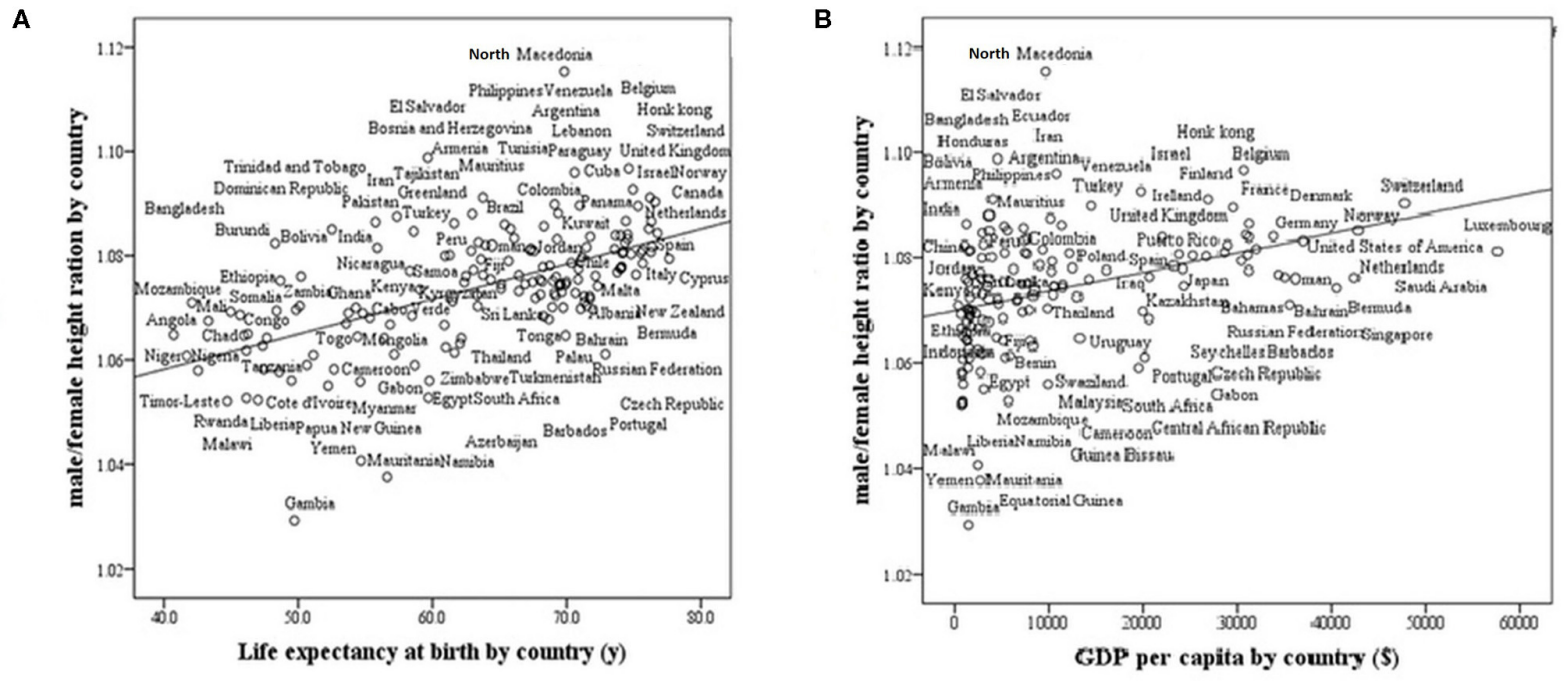
Male/female height ratio as a function of life expectancy at birth and GDP per capita in 161 modern countries. R-Pearson's correlation coefficient. **(A)** Life Expectancy at birth by country. *R* = 0.57, *p* < 0.0001. **(B)** GDP (Gross domestic product based on purchasing power parity) per country. *R* = 0.40, *p* < 0001.

We found that life expectancy at birth and GDP strongly and positively correlate with the M/F height ratio in modern countries (*r* = 0.57, *p* < 0001; *r* = 0.40, *p* < 0001, respectively, Figure 3). The ratio is greater in countries with high life expectancy at birth (healthy countries) and in countries with a high GDP (wealthy countries).

**Figure 4:**
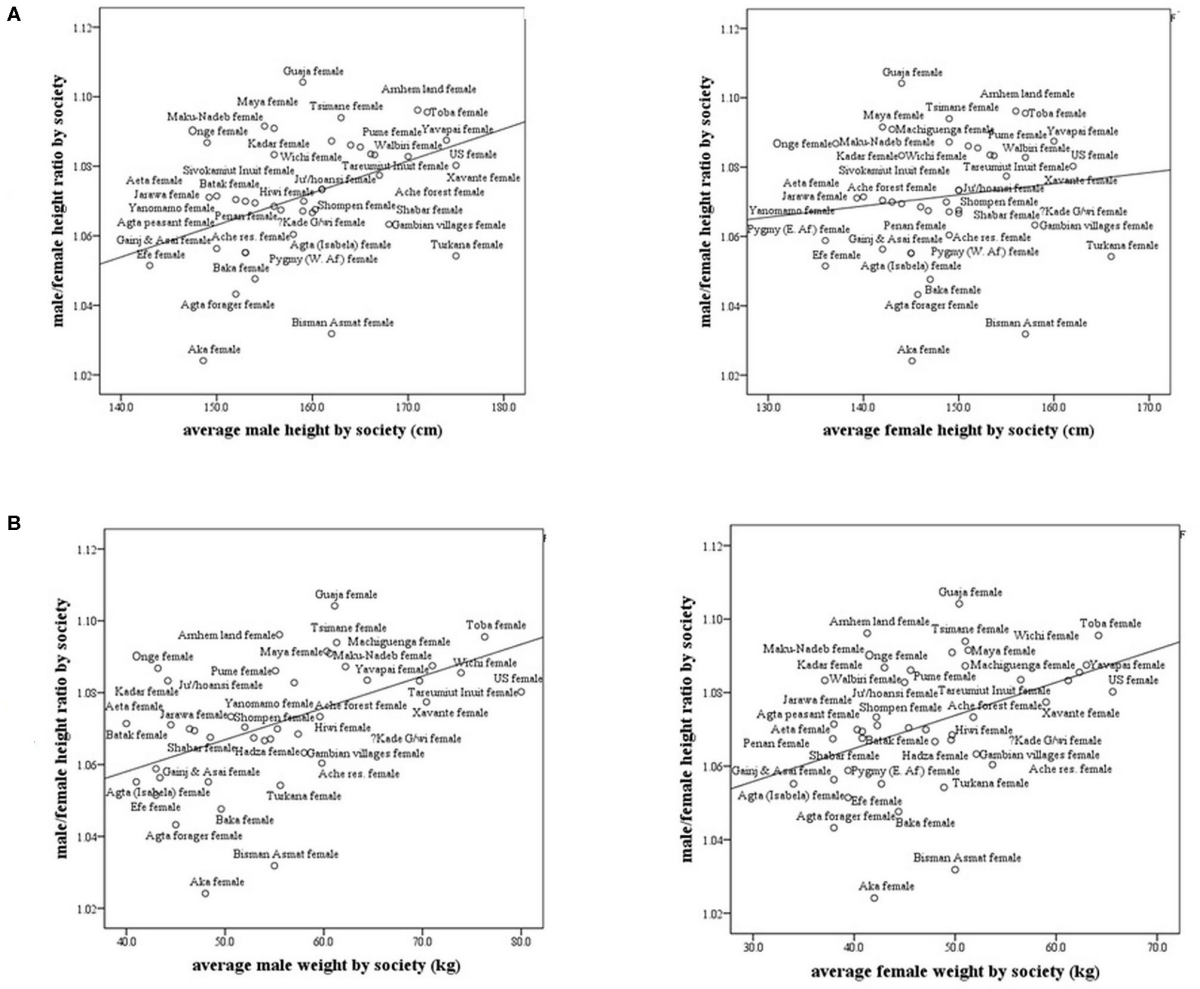
Male/female height ratio as a function of the average male and female height and weight in subsistence-based preindustrial societies. R-Spearman's correlation coefficient. **(A)** Male/female height ratio as a function of the average male and female height in subsistence-based preindustrial society. For males, *R* = 0.41, *p* < 0.007 and for females, *R* = 0.15, *p* = 0.33. **(B)** Male/female height ratio as a function of the average male and female weight by society. For males, *R* = 0.57, *p* = 0.0001 and for females, *R* = 0.43, *p* = 0.005.

In subsistence-based preindustrial societies, we also found that some allometry occurs. Specifically, we found that the M/F height ratio correlated positively and strongly with the male and female weight (*r* = 0.57, *p* < 0.0001; *r* = 0.43, *p* < 0.001, respectively) and with male height only (*r* = 0.41, *p* < 0.001), (Figures 4A,B). The correlations with weight were stronger than that for height. These results found in preindustrial societies are similar to those found in modern countries, the correlation between male/female height ratio with male size was greater than with the female size (Tables 1, 2). In preindustrial societies, male and female height and weight correlated strongly and significantly with life expectancy at birth and at age 15 years (Table 3).

**Table 1:** Analysis of allometry for 161 modern countries.

	**Male**	**Female**
Male/Female height ratio correlated with height per country *(R)*	0.52***	0.18**
Male/Female height ratio correlated with weight per country *(R)*	0.54***	0.29***

*Sexual dimorphism in body size as a function of the average male and female height and weight*.

*R-Pearson's correlation coefficient*.

**p < 0.001, ***p < 0.0001.

**Table 2:** Analysis of allometry for 38 subsistence-based preindustrial societies.

	**Male**	**Female**
Male/Female height ratio correlated with height *(R)*	0.41**	0.15^NS^
Male/Female height ratio correlated with weight *(R)*	0.57***	0.43**

*Sexual dimorphism in body size as a function of the average male and female height and weight*.

R-Spearman's correlation coefficient, **p < 0.001, ***p < 0.0001.

*NS, not significant*.

**Table 3:** Male and female height and weight correlated with life expectancy at birth and at age 15 y and population density in subsistence-based preindustrial societies.

	**Life expectancy at birth (R)**	**Life expectancy at age 15 y (R)**	**Density population, (R)**
Average male height	0.69**	0.61**	NS
Average female height	0.72**	0.70**	NS
Average male weight	0.8**	0.68**	−0.45*
Average female weight	0.71**	0.67**	−0.48**

*R-Spearman's correlation coefficient*.

*p < 0.01. **p < 0.001.

## Discussion

In *Homo* species and the great apes, males have greater body size and are more muscular and stronger than females (3, 14). As well as these biological differences between males and females, other determinants, such as nutrition, health care, and disease, might play an important role in determining height differences between males and females. Here, we report that the ontogeny of size dimorphism is related to sex hormones; it is evident during minipuberty, and it is established during puberty. It is greater in tall populations living in good economical and health circumstances than in short populations living in poor and stressful conditions.

Gray and Wolf and others found that dietary factors can influence the degree of sexual dimorphism in size resulting in a decrease in male-female height differences under conditions of nutritional stress and an increase the M/F height ratio with improved diet (15–18).

We found a positive correlation between the M/F height ratio and health, as indicated by life expectancy. It seems that women are more resilient during environmental stress (19). This summary of a sex difference in mortality included the probability of survival to age 70 by county in the United States, the Human Mortality Database data for 18 high-income countries since 1900, and mortality data within and across developing countries over time periods for which reasonably reliable data are available. These data reveal that in each period of economic development after the onset of demographic and epidemiologic transition, cross-sectional variation in sex differences exhibits a consistent pattern of female resilience to mortality under adversity.

Current theory on the sexual dimorphism of the stress response posits that females exhibit an affiliative behavior (a “tend and befriend” response) whereas males exhibit more of a fight or flight response to stress (20). Oxytocin is thought to be important in female affiliative behavior whereas testosterone or vasopressin might be more important for male social behaviors (21). In a US national sample of 432 female and 1,200 male Vietnam veterans, for both genders direct links to post-traumatic stress disorder PTSD from war-zone, war-zone stressors appeared preeminent for PTSD in men, while post-trauma resilience recovery variables were more salient for women (22). This sexual dimorphism in the stress response is supported by experimental work with rodents. In a study of prenatal restraint (PS) stress on anxiety- and depression-related behavior in both male and female adult Sprague-Dawley rats, PS significantly increased anxiety-related behavior in male, but not in female offspring. Likewise, depression-related behavior increased in male PS rats only. Further, male PS offspring showed increased basal plasma corticosterone levels in adulthood (23). Further, the data indicated that epigenetic regulation is affected differentially in male and female PS offspring. These sex-specific alterations may, at least in part, explain the female resilience hypothesis. The Chinese Center for Disease Control and Prevention published an analysis of coronavirus cases. Although men and women have been infected in roughly equal numbers, the death rate among men was 2.8 percent, compared with 1.7 percent among women. Men also were disproportionately affected during the SARS and MERS outbreaks (24). One hypothesis is that women's stronger immune systems confer a survival advantage to their offspring, who imbibe antibodies from mothers' breast milk that help ward off disease, while the infants' immune systems are still developing (24). It was suggested that estradiol also contributes to cognitive stress resilience in females (25). The contribution of this mechanism, combined with intra-hippocampal synthesis of estradiol, contributes to mediating cognitive resilience to chronic stress demonstrated by females, but not males.

Moreover, males are more susceptible to fluctuations in nutritional quality; for example, the impairment in long bone growth is greater in males than females under the same food deficits (14). However, food consumption behavior cannot be neglected at higher income levels; for example, teenage girls in wealthy societies in the twenty-first century might consume less food in order to be slim for cultural and fashion reasons. Before the industrial revolution, food, and health resources were scarce, and there were gender-related conflicts about its distribution. A long-term nutritional shortage and poor health during childhood can result in a reduced adult height in both sexes with greater impact on men's height than that of women.

In this investigation, we defined sexual dimorphism in body size as the ratio between male and female height. This definition differs from that of some biologists and anthropologists who used the difference between the mean heights of males and females to define sexual dimorphism in body size. We also used national averages in height and weight, while recognizing variations within countries. We previously found that between-countries variations are somewhat smaller than within countries: for men, the between-countries average height ranges from 158 to 183 cm (Indonesia and Netherlands, respectively), with a standard deviation (*SD*) of 5.9 cm, as compared to 164–190 cm with an *SD* of 6.5 cm within the USA (CDC 2000; 3–97th percentile).

Gray and Wolfe (16) compared the mean heights of men and women in various societies from Africa, the Mediterranean region, Eastern Eurasia, and North and South America. The results of our study confirm their results of increasing sexual dimorphism in body size with increasing height of the population. Baten and Murray performed a time-series analysis by birth cohort of average heights of men and women who were imprisoned in nineteenth century in Bavaria (26) They showed that final adult height of women responded much more systematically than did men's heights to differences in economic, nutritional and disease conditions in infancy. Female height, but not male height, was reduced by the 1840 potato crisis, tuberculosis prevalence, and illegitimate birth. Finally, a study of the Indian population between the 1930s and 1970s reported that the mean height of both females and males increased when the food supply increases in a similar manner. However, they also reported an increase in sexual dimorphism in body size that occurred during a food crisis in the states of Kerala and Orissa when a clear decline in height of both men and women was evidenced, pointing in their view to a rise in female discrimination during poor times when care and investment in girls is reduced disproportionately (27).

Gustafsson and Lindenfors investigated whether there was an allometric relationship between male and female stature in humans in 124 populations who lived in the latter part of the twentieth century. They found that sexual dimorphism in body size did not correlate with height (28). Here, we describe a strong hyperallometry when the M/F height ratio is correlated with male, but not female, body size. Hence, we posit that sexual dimorphism in body size can be attributed to a male's negative response to a stressful environment.

Holden and Mace investigated the connection between sexual dimorphism in body size and the division of labor in worldwide sample of 76 nonindustrial populations (29). They used data on sexual division of labor for five subsistence activities, namely hunting, gathering, fishing, pastoralism, and agriculture, from an “Ethnographic Atlas” by Murdock 1967, data on stature were taken from a variety of published sources. They concluded that sexual dimorphism in stature is negatively correlated with the extent of women's participation in the labor force and a sex-biased parental investment. They also found that women were taller than men in those societies, that the women's contribution to food production was greater than that of the men resulting in improvement of females' nutritional status in these societies.

Other factors which have been reported to influence the M/F height ratio in preindustrial societies are probably the male and female division in agricultural tasks (30). The women in these societies usually were engaged in cattle farming and garden work, thereby increasing their “advantage of proximity” to milk and vegetables. In contrast, grain cultivation which was usually done by men requires more upper-body strength than herding cattle; hence a grain-oriented society might distribute more nutritional and health resources to male offspring (3). When agricultural patterns changed, for example from cattle farming to grain-based agriculture, women's height, and health might have declined (31).

Finally, the calculation of target height and the prediction of adult height are influenced by the sexual difference in height in various countries. The method of calculating the mid-parental target height was developed in the nineteenth century in the United Kingdom: the mean sex height difference was typically 13 cm; and we therefore used the formula: (father's height + mother's height + 13 cm)/2 for boys and (father's height + mother's height −13 cm)/2 for girls. We now show that the average male/female height ratio and also the mean sex difference in height are dissimilar in different countries (Figures 2, 3). In the calculation of mid-parental height and target height the male-female difference for each country needs to be adjusted to a country's sexual dimorphic size ratio.

The utilization of anthropometric data by country bears a certain limitation. The population of some countries descent from many different immigrations arrived in different moments at the same country. Averaging gives extra weight to that majority ethnic group of a country.

We conclude that sexual dimorphism in body size occurs when (a) the growth velocity is maximal during infancy and adolescence, (b) living standards are high, and health correlate positively with male/female height ratio. Anthropological studies and our results emphasize mostly the female resiliency hypothesis: shorter male heights in times of environmental stress lead to smaller sexual dimorphism in body size.

## Data Availability Statement

All datasets generated for this study are included in the article.

## Author Contributions

AG co-conceptualized and co-designed the study, carried out the statistical analysis and interpreted the results, and reviewed, revised and approved the study. ZH conceptualized and designed the study, oversaw it's conduct and drafted the initial manuscript, and reviewed, revised, and approved the study. Both authors contributed to the article and approved the submitted version.

## Conflict of Interest

The authors declare that the research was conducted in the absence of any commercial or financial relationships that could be construed as a potential conflict of interest.
